# Thirdhand Smoke in Review: Research Needs and Recommendations

**DOI:** 10.1289/ehp.119-a399b

**Published:** 2011-09-01

**Authors:** Tanya Tillett

**Affiliations:** Tanya Tillett, MA, of Durham, NC, is a staff writer/editor for *EHP*. She has been on the *EHP* staff since 2000 and has represented the journal at national and international conferences.

Studies over the last half-century have clearly demonstrated that cigarette smoking is associated with adverse health effects both for smokers and for individuals exposed to secondhand smoke (SHS). Now a new level of exposure has been identified: thirdhand smoke (THS), or residual tobacco smoke pollutants that remain on surfaces and in dust and that are reemitted in the gas phase and interact with other compounds. In a new review, researchers offer a descriptive analysis of THS constituents and dynamics and argue for the establishment of a programmatic research agenda to close gaps in our understanding of the nature and effects of THS [*EHP* 119(9):1218–1226; Matt et al.].

THS exposure is the result of inhalation, ingestion, or dermal uptake of THS pollutants in the air, in dust, and on surfaces. The authors point out that THS and SHS are closely related, and in fact coexist as THS is first formed and in settings where smoking recurs regularly. But whereas SHS is removed by ventilation, THS pollutants may persist in environments for several hours or days after tobacco has been smoked.

THS components—such as nicotine and carcinogenic polycyclic aromatic hydrocarbons, including benzo[*a*]pyrene—are sorbed and reemitted from indoor surfaces over varying periods of time after tobacco sources have been extinguished. Some THS components react with other environmental compounds to produce secondary pollutants. For instance, chemical reactions between nicotine and nitrous acid lead to the formation of additional tobacco-specific nitrosamines, and ozone can react with certain volatile organic compounds to form formaldehyde, acetaldehyde, and benzaldehyde.

**Figure:**
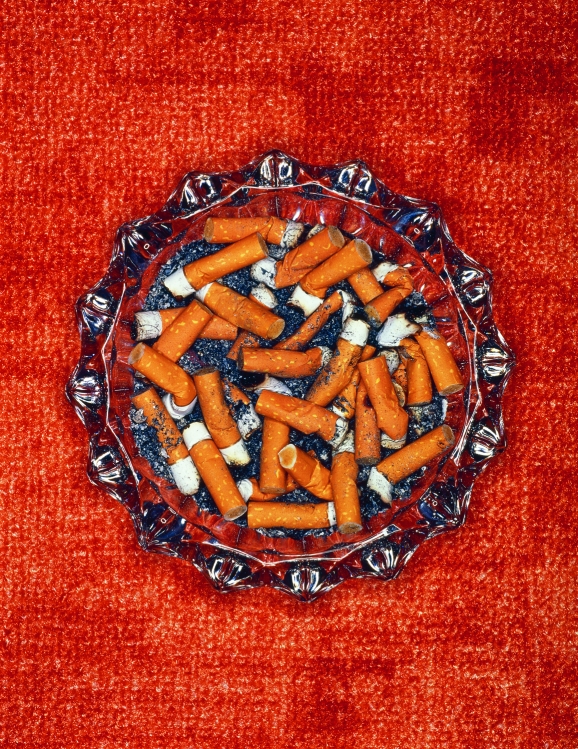
Thirdhand smoke lingers after secondhand smoke has cleared from the room. T. Hoenig/A.B./Media Bakery

THS constituents have been measured in indoor spaces months after smoking last occurred. Given the U.S. Surgeon General’s conclusion that there appears to be no risk-free level of exposure to SHS, this raises the possibility that THS could lead to potentially harmful exposures, particularly for vulnerable populations such as children. The investigators recommend that future studies further define the chemistry and toxicology of THS, which will aid in establishing, enhancing, and enforcing public policies and personal practices that limit tobacco smoke exposure.

The authors note that voluntary public and private policies enacted over the last decade—including an increase in smoke-free workplaces, public areas, and lodging—have effectively addressed THS exposure in some settings. They argue that stronger public health policies can be developed through an interdisciplinary research agenda that combines basic and applied risk assessment research with studies on tobacco use and cessation.

